# Rise of biologics in noninfectious uveitis: a retrospective cohort study from Nepal

**DOI:** 10.1097/MS9.0000000000000546

**Published:** 2023-04-11

**Authors:** Sadhana Sharma, Ranju Kharel, Sanket Parajuli, Saket Jha

**Affiliations:** a B.P. Koirala Lions Center for Ophthalmic Studies (BPKLCOS), Institute of Medicine; b Department of Internal Medicine, Maharajgunj Medical Campus, Institute of Medicine, Kathmandu; c Reiyukai Eiko Masunaga Eye Hospital, Banepa, Nepal

**Keywords:** noninfectious uveitis, biologics, scleritis, tofacitinib

## Abstract

Uveitis is a sight-threatening disease that poses a heavy burden on the quality of life. The treatment of uveitis has been revolutionized in the past two decades. Most remarkable among these is the emergence of biologics, which have shown to be effective and safer therapeutic option in noninfectious uveitis. Biologics are very useful when conventional immunomodulator therapy has failed or has been poorly tolerated. The most widely used biologics are tumor necrosis factor-α inhibitors (infliximab and adalimumab) with promising results. Other drugs include anti-CD20 inhibitors (rituximab), interleukin-6R-inhibitor (tocilizumab), interleukin-1R-inhibitor (anakinra), and Janus-associated kinase inhibitor (tofacitinib)

**Methods:**

A retrospective review of all cases of noninfectious uveitis and scleritis presenting to our center from July 2019 to January 2021 and had been treated with biological therapy were included.

**Results:**

We included 12 eyes of 10 patients. The mean age was 42.10±9.71 years. Anterior nongranulomatous uveitis comprised 70% of the cases and the most common etiology of anterior uveitis was spondyloarthritis (seven cases among which five cases were nonradiographic) axial spondyloarthritis (human leukocyte antigen B27 positive) followed by radiographic axial spondyloarthritis (two cases). The first line of treatment in all cases was conventional synthetic disease-modifying antirheumatic agents among which 50% (n=5) had received methotrexate (≥15 mg/week). As a second line of treatment, one or more biologics was used. Majority of the patients received oral tofacitinib 50% (n=5) followed by Inj adalimumab 30% (n=3). One case of Behcet’s disease required sequential biologics (Inj adalimumab followed by oral tofacitinib). All patients tolerated and responded well to the treatment and no recurrences were observed after discontinuation of biologics drugs during the follow-up period of 1 year.

**Conclusion:**

Biologics are a relatively safe and effective modality of treatment in refractory, recurrent noninfectious uveitis.

## Introduction

Highlights

- Biologic drugs are effective therapeutic alternative for management of refractory cases of noninfectious uveitis (NIU).
- Risk–benefit assessment is of paramount importance before starting biologics.
- Multidisciplinary approach with co-management by uveitis specialist and rheumatologist is preferred.

Uveitis includes a group of heterogeneous diseases, primarily involving the uveal tract with or without the involvement of the adjacent intraocular structures. It is an important cause of vision loss accounting for ∼25% of total blindness in developed countries and 25% of blindness in developing countries^1^,^2^. Based on etiology, uveitis may be classified as infectious or noninfectious (autoimmune disorders). In the working-age population of developed countries, NIU is the leading cause of irreversible blindness^1^.

Systemic autoimmune disease that may cause uveitis includes inflammatory diseases–such as rheumatoid arthritis, spondyloarthropathies, inflammatory bowel disease, Behçet’s disease (BD), sarcoidosis, or juvenile idiopathic arthritis (JIA), and autoimmune diseases that preferentially affect ocular tissues includes Vogt–Koyanagi–Harada, white dot syndromes, sympathetic ophthalmia, birdshot chorioretinopathy, etc.^1^.

The goal of treatment in uveitis is to control inflammation, preserve vision, prevent recurrences as well as minimize the adverse effects of medications.

The first line of drugs in NIU are corticosteroids due to their high efficacy and rapid onset of action but long-term use is limited due to multiple side effects. Immunomodulators are used in chronic NIU refractory/dependance to steroids or as a steroid-sparing agent. Immunomodulatory therapy can be divided into conventional synthetic, targeted synthetic, and biologics. Biologic response modifiers are a category of immunomodulatory drugs that are either manufactured, extracted from, or semisynthesized from a biologic source and target inflammatory molecules, such as cytokines. They are used in place of, or concomitant with, other immunomodulatory drugs^3^ and are widely used for NIU over the past decade. Biologics used in uveitis comes in a variety of forms, including monoclonal antibodies, bioengineered receptor complexes, cytokine antagonists, or cytokines^4^ The commonly used biologics for uveitis are tumor necrosis factor-α inhibitors, Interleukin inhibitors and janus kinase inhibitors.

In this study, we aim to evaluate the use of different biologic agents in the treatment of refractory NIU and scleritis among Nepalese patients. This is the first report from Nepal documenting the use of biologics for ocular inflammatory conditions.

## Methods

A hospital-based, retrospective study was conducted. Data of all refractory cases of uveitis and scleritis, who received treatment with biological agents from 2019 to 2021 were retrieved from the medical record section. The study was approved by the Institutional Review Committee (IRC) and adhered to the Declaration of Helsinki.

A detailed history was taken in each patient including demographic details, course of the disease, prior similar episode, recurrences, and history of tuberculosis or concomitant systemic diseases. Also, drug history and side effects were noted. In all patients, best-corrected distant visual acuity, near vision, and intraocular pressure measurement using Goldmann applanation tonometry, slit-lamp examination, and indirect ophthalmoscopy were performed at baseline and all follow-up visits. Routine laboratory investigations included a complete blood count, erythrocyte sedimentation rate, C-reactive protein, Mantoux test, chest x-ray, urine routine, and microscopic examination, serum calcium, serum uric acid, rheumatoid factor, serologies for HIV, hepatitis B surface antigen, hepatitis C antibody, Veneral Disease Research Laboratory (VDRL) for syphilis. Additional investigations were advised whenever necessary.

The uveitis was classified as per the criteria of the Standardization of Uveitis Nomenclature Working Group (SUN). Remission was defined as an inactive disease for at least 3 months and relapse was considered when a patient who was in remission experienced a new flare of uveitis.

Refractory uveitis/scleritis was defined as relapse within 3 months despite receiving corticosteroids (10 mg/day or more) as maintenance therapy and/or with one or more immunosuppressive(s).

All the patients were routinely evaluated and investigated by a single uveitis specialist and active infection was ruled out. In cases with clinical suspicion of tuberculosis, sarcoidosis, referral was made to the pulmonologist. Based upon the uveitis status, the decision to initiate biological therapy was done by the uveitis specialist after consultation with the immunologist. The selection of biologics, duration of therapy, and monitoring of the systemic side effects was done by the immunologist in consultation with the uveitis specialist. This study has been reported in line with the STROCSS criteria^5^.

## Results

Twelve eyes of 10 patients who received biologics therapy between July 2019 and January 2021 were enrolled. The mean age was 42.10±9.71 years with male:female ratio of 1.2:1 and the details of the demographic clinical profile of the patients are shown in Table 1.

**Table 1:** Clinical profile of the patients

	*n* (%)
Number of patients (eyes)	10 (12)
Age (mean±SD)	42.10±9.71
Male/female	6/4
Laterality (unilateral/bilateral)	8/2
Etiological diagnosis	
Spondyloarthritis (radiolographic and nonradiographic)	7 (50)
Behcet’s disease	1 (10)
Wegener’s granulomatosis	1 (10)
Anatomical diagnosis	
Anterior uveitis	7 (70)
Intermediate uveitis	2 (20)
Scleritis	1 (10)

**Figure 1:**
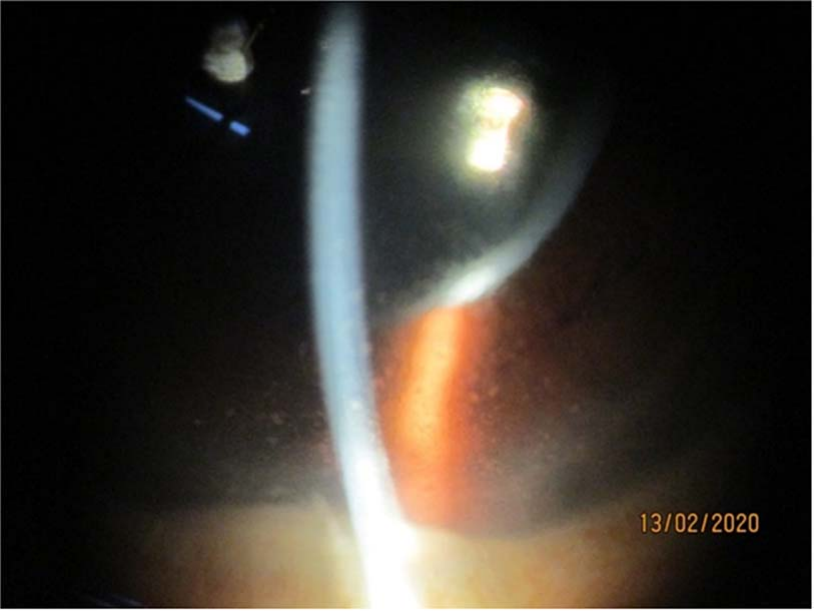
A case of acute anterior uveitis (human leukocyte antigen B27 positive) presenting with hypopyon.

**Figure 2:**
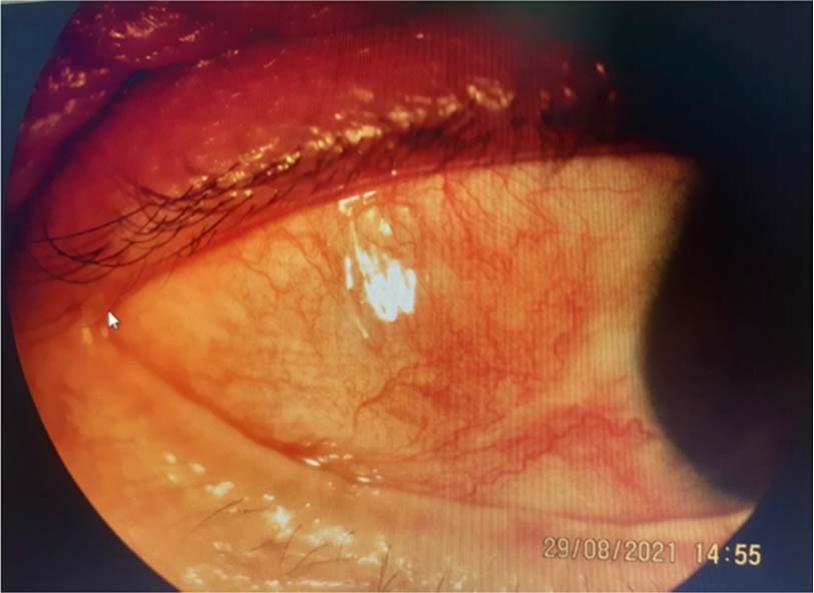
A case of anterior non-necrotizing scleritis in a case of Wegener’s granulomatosis.

Anterior uveitis (nongranulomatous) comprised 70% (seven cases) (Fig. 1) followed by 20% (2 cases) of intermediate uveitis and 10% (one case) of recurrent scleritis (Fig. 2). Most common etiology of anterior uveitis was spondyloarthritis (seven cases among which five cases were nonradiographic axial spondyloarthritis (HLAB27 positive) followed by radiographic axial spondyloarthritis (two cases). The one case of intermediate uveitis was due to BD and the case of recurrent scleritis was due to Wegener’s granulomatosis.

First line of treatment in all cases was conventional synthetic disease-modifying antirheumatic agents. Five cases (50%) had received methotrexate (≥15 mg/week), two cases (20%) received sulfasalazine (≥500 mg b.i.d.), and one case (10%) received cyclophosphamide as a first line of steroid-sparing agent. Two patients (20%) were on dual immunosuppressives (methotrexate and sulfasalazine) before switching to biological therapy.

Indication to switching to second-line drugs were poor response in seven cases, allergy to sulfasalazine in two cases, and side effects (oral ulcers) due to Methotrexate in one case. Mean duration between diagnosis of uveitis and initiation of immunosuppressive therapy before planning biologics drugs was 12.52 months (range: 3–18 months). Mean duration between switching of the conventional drugs to biologics was 2.5 months (range: 1–3 months).

After switching to the biological therapy, majority patients received oral tofacitinib (50%) followed by Inj adalimumab (30%) as a second line of treatment, whereas the case of scleritis was treated with Inj rituximab. One case of BD required sequential biologics (Inj adalimumab followed by oral tofacitinib) for control of inflammation.

The mean dose of corticosteroids could be reduced to 5 mg/day when used alongside biological drugs.

All of the patients maintained stable visual acuity and adequate control of inflammation with the biologic drugs. No side effects associated with biologics were seen. Furthermore, no recurrences were observed after discontinuation of biologic drugs during the follow-up period.

The details of our patients under biological therapy are presented in Table 2.

**Table 2:** Details of the patients under biologics

Patient no.	Age/sex	Etiology of uveitis	Presentation	First line of steroid-sparing agent	Biologics	Reason for switching
1	47/F	Nonradiographic axial spondyloarthritis (HLAB27 positive)	RE recurrent anterior uveitis	Sulfasalazine 500 mg b.i.d.	Tofacitinib 5 mg p.o. b.i.d. ×4 months	Poor response
2	34/M	Radiographic axial spondyloarthritis	RE recurrent anterior uveitis	Methotrexate 15 mg/week	Inj adalimumab 40 mg s.c. monthly ×4 months	Poor response
3	29/F	Nonradiographic axial spondyloarthritis (HLAB27 positive)	LE recurrent anterior uveitis	Methotrexate 15 mg/week	Tofacitinib 5 mg p.o. b.i.d. ×7 months	Side effects (oral ulcers)
4	43/M	Behcet’s disease	B/L intermediate uveitis	Methotrexate 15 mg/week	Inj adalimumab 40 mg s.c. ×10 months Tofacitinib 5 mg p.o. b.i.d. ×3 months	Poor response
5	46/M	Nonradiographic axial spondyloarthritis (HLAB27 positive)	LE anterior uveitis	Methotrexate 15 mg/week	Inj adalimumab 40 mg s.c. monthly ×5 months	Poor response
6	32/M	Radiographic axial spondyloarthritis	RE anterior uveitis	Methotrexate 15 mg/week Sulfasalazine 500 mg b.i.d.	Inj adalimumab 40 mg s.c. monthly ×3 months	Poor response
7	35/F	Nonradiographic axial spondyloarthritis (HLAB27 positive)	RE anterior uveitis	Sulfasalazine 500 mg b.i.d	Tofacitinib 5 mg p.o. b.i.d. ×5 months	Allergy to sulfasalazine
8	55/F	Wegener’s granulomatosis	LE recurrent anterior scleritis	Cyclophosphamide	Inj rituximab 1000 mg i.v. 6 months	Poor response
9	58/M	Idiopathic	BE intermediate uveitis	Methotrexate 15 mg/week Sulfasalazine 1000 mg t.i.d	Tofacitinib 5 mg p.o. b.i.d. ×4 months	Poor response
10	42/M	Nonradiographic axial spondyloarthritis (HLAB27 positive)	LE anterior uveitis	Methotrexate 15 mg/week	Tofacitinib 5 mg p.o. b.i.d. ×3 months	Poor response

BE, both eye; B/L, Bilateral; F, female; HLA, human leukocyte antigen; LE, left eye; M, male; RE, right eye.

## Discussion

Adalimumab is the first and only Food and Drug administration (FDA) approved steroid-sparing drug for NIU in adults. A meta-analysis of six randomized controlled trials (605 patients ) systematically reviewed the efficacy and safety of Adalimumab in NIU results of which showed an almost 50% decrease in the risk of treatment failure in NIU^6^.

Rituximab shows good response for scleritis associated with granulomatosis with polyangiitis and rheumatoid arthritis, uveitis associated with JIA, Vogt–Koyanagi–Harada disease, BD with some reports suggesting the drug to be more efficacious for cases of noninfectious scleritis than for uveitis^7^.

Other group of biologics are Janus-associated kinase inhibitors, a long-term oral treatment options of rheumatoid arthritis. Tofacitinib being a small molecule, may efficiently cross the blood-aqueous barrier to exert its anti-inflammatory effects^8^. Tofacitinib has been reported to be useful in controlling recurrences of human leukocyte antigen (HLA) B27-associated uveitis, rheumatoid arthritis, psoriatic arthritis, and ulcerative colitis^9^.

Tofacitinib reported to be effective in an adult JIA patient with anterior uveitis and macular edema^10^, a patient with scleritis and a patient with anterior and intermediate uveitis^11^. In our study, majority of the patients received tofacitinib and responded well to the treatment.

Similar study by Sadhu *et al.* 12 reported use of four biologic agents – infliximab, adalimumab, rituximab, and golimumab in refractory scleritis and uveitis. Adalimumab was the most common (61%) biological drug used followed by infliximab. Recurrence was seen in three cases who received adalimumab, two of them developed recurrence 6 months after stopping the drug and one had recurrence while on adalimumab.

In the current study, biological therapy showed a good clinical response in treatment resistant cases previously treated with conventional immunomodulators. This is the first-ever reported study on biologics in uveitis from Nepal. However, due to the retrospective design, limited number of cases, and short-term follow-up, the findings of this study cannot be generalized.

## Conclusions

Biologic drugs are increasingly being used for vision-threatening, NIU, and scleritis in the past decade. Although evidences suggest biologics to be a safe and effective option, these are not free of side effects. So, patients must be carefully monitored for potential side effects, most specifically infections and demyelinating diseases. In tuberculosis endemic country like ours, these agents should be used with utmost caution^12^. Multidisciplinary approach preferably among uveitis specialist and rheumatologist can help manage comorbidities effectively, minimize complications and thus result in best outcome.

## Ethical approval

Approved by Institutional Review Committee (IRC) (IRC no: 116(6-11) E2).

## Consent

Written consent was taken from all the patients.

## Sources of funding

None.

## Author contribution

S.S.: study concept/design, data collection, data analysis, and preparation of manuscript. R.K.: study concept/design, data collection, and manuscript revision. S.P.: study concept, data analysis, and review of manuscript. S.J.: study concept and review of manuscript. All authors approved the final manuscript.

## Conflict of interest

The authors declare that they have no conflicts of interest.

## Research registration unique identifying number (UIN)

1. Name of the registry: Institutional Review Committee (IRC) of Institute of Medicine, Maharajgunj, Kathmandu, Nepal
2. Unique Identifying number or registration ID: 116(6-11) E2)
3. Hyperlink to your specific registration (must be publicly accessible and will be checked)

## Guarantor

Sadhana Sharma.

## Provenance and peer review

Not commissioned, externally peer reviewed.
